# Controlling the Synchronization of Molecular Oscillators through Indirect Coupling

**DOI:** 10.3390/mi13020245

**Published:** 2022-02-01

**Authors:** Shiho Inagaki, Nathanael Aubert-Kato

**Affiliations:** Department of Information Sciences, Ochanomizu University, Tokyo 112-8610, Japan; inagaki.shiho@is.ocha.ac.jp

**Keywords:** molecular robotics, repressilator, synchronization, enzymatic coupling

## Abstract

In this article, we study the coupling of a collection of molecular oscillators, called repressilators, interacting indirectly through enzymatic saturation. We extended a measure of autocorrelation to identify the period of the whole system and to detect coupling behaviors. We explored the parameter space of concentrations of molecular species in each oscillator versus enzymatic saturation, and observed regions of uncoupled, partially, or fully coupled systems. In particular, we found a region that provided a sharp transition between no coupling, two coupled oscillators, and full coupling. In practical applications, signals from the environment can directly affect parameters such as local enzymatic saturation, and thus switch the system from a coupled to an uncoupled regime and vice-versa. Our parameter exploration can be used to guide the design of complex molecular systems, such as active materials or molecular robot controllers.

## 1. Introduction

In the past two decades, advances in synthetic biology have provided researchers with the ability to implement complex dynamical systems both in vivo [1,2] and in vitro [3,4,5,6].

**Figure 1:**
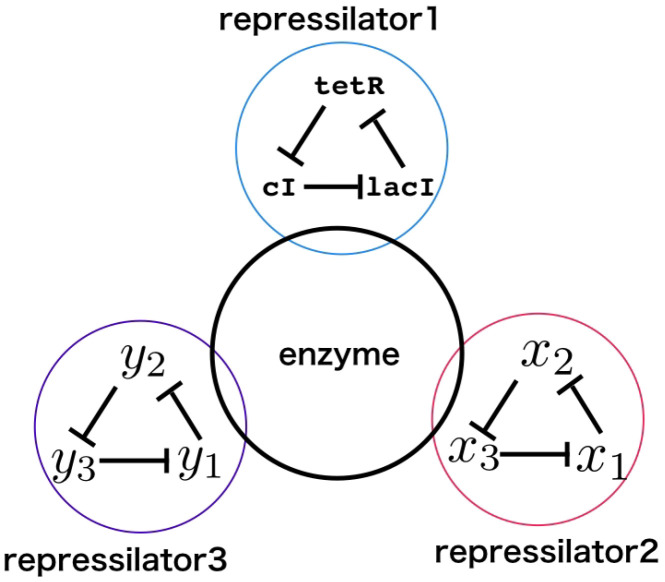
General design of the system: three identical reaction networks (repressilators) are implemented with independent molecular species, while sharing the same enzymes. Each network is made of three species being continuously produced by the system while repressing the creation of the next species in the cycle. Direct interactions are prevented and coupling can only occur through competition for enzymatic resources.

Among those dynamical systems, particular focus has been given to oscillators, both for their complex behaviors [1,7] and their biological relevance [8,9,10,11,12]. In this paper, we focus on one of the most emblematic synthetic oscillators, called the repressilator [1], consisting of a cycle of three negative feedbacks (Figure 1).

The effect of direct coupling between repressilators through communication modules has been studied previously [13], and can lead to partial synchronization or even chaos [7]. In such cases, identical replicates of repressilators are localized in different cells and communicate through the diffusion of molecular species through the membrane. However, we can design a molecular system with multiple instances of a repressilator locally. Thanks to the abstraction of the oscillatory mechanism, we can implement multiple such oscillators based on independent molecular species following the same reaction network [5]. In that case, while no direct interaction should be observed, those oscillators will remain indirectly coupled through enzymatic saturation [14]. Research in the field of molecular robotics usually studies such enzymatic saturation with the goal of avoiding it. Lower saturation usually means that the system behaves in a simpler way, making it easier to design and manipulate. However, we believe that the non-linearity introduced by such coupling is important, as it can potentially increase the behavior space of a given system, which is otherwise limited by the design space of molecular species.

As such, we chose to use a scanning strategy to explore the impact of indirect saturation on a system of three indirectly coupled repressilators (Figure 1). We extended the model of the repressilator to include indirect saturation, taking inspiration from Rondelez’s model [15]. We used an extended definition of autocorrelation [16] to determine the overall coupling in the system and found regions of the (concentration, enzymatic saturation) space with quick transitions between completely coupled, partially coupled, and uncoupled states.

Additionally, such coupling can be tuned over time, as it will depend on the saturation level and activity of the enzymes, and thus it can be used as a control mechanism for the system [17]. As periodic behaviors are typical in the control of the behavior of molecular robots (see for instance [18]), mapping the behavior space of a multi-oscillator system is a first step in designing complex dynamic controllers for such systems. Molecular oscillators have also been studied as a fundamental part of morphogenesis. For instance, the clock and wave-front mechanism, in which the state of a local molecular clock is “locked in place” by a traveling wave, provides spatial differentiation [19,20]. Taking inspiration from that mechanism, synthetic oscillators can be applied to the study of morphogenesis [21] and to the creation of novel active materials [22].

## 2. Method

### 2.1. Model

In the original implementation of the repressilator, the genes lacI, tetR, and cI express their respective protein and inhibit the transcription of the gene tetR, cI, and lacI, respectively. As such, when lacI is being expressed, tetR is being inhibited, meaning that its protein is being degraded over time, thus freeing cI from inhibition. Once that inhibition is low enough, cI will be expressed, thus eventually inhibiting lacI. In turn, tetR will eventually stop being inhibited and start inhibiting cI, completing the cycle.

The original repressilator mechanism can be modeled as:

(1) $$\begin{array}{cc} \; & \frac{dm_{i}}{dt}=-m_{i}+\frac{\alpha }{1+p_{i-1}^{n}}+\alpha_{0}\\ \; & \frac{dp_{i}}{dt}=-\beta \left(p_{i}-m_{i}\right). \end{array}$$

Here, $m_{i}$ and $p_{i}$ are proportional to mRNA and protein concentrations. The second term on the right side in the above expression reflects the synthesis of the mRNAs from the DNA. *n* is the cooperativity of repression [23]. $\alpha_{0}$ and α are the transcription rate of a repressed promoter and of a free promoter. β is the ratio of protein and mRNA decay rate [24]. Next, we extent that model to three oscillators with shared enzymatic access. The system thus becomes:

(2) $$\begin{array}{cc} \; & \frac{dm_{i,j}}{dt}=\frac{\alpha_{i}}{1+p_{i,j-1}^{n}}+\alpha_{0,i}-\frac{km_{i,j}}{K_{m}+\sum_{r,s}m_{r,s}}\\ \; & \frac{dp_{i,j}}{dt}=\beta_{i}m_{i,j}-\frac{kp_{i,j}}{K_{p}+\sum_{r,s}p_{r,s}} \end{array}$$

$m_{i,j}$ and $p_{i,j}$ are the concentration of the *j*th mRNA and protein of repressilator *i*, respectively. In this model, the degradation term for $m_{i,j}$ now follows Michaelis-Menten dynamics with coupled enzymatic saturation. Competition with other substrates is indicated as a sum of concentrations in the denominator [15]. *k* is the maximum rate of degradation. $K_{m}$ and $K_{p}$ are the Michaelis constants of mRNAs and proteins, respectively. To keep the model simple, we assume that *k* for the degradation of mRNAs and *k* for the degradation of proteins have the same value. That assumption is based on the fact that their respective value are close enough in the original model [1]. Furthermore, those values can be adjusted through tuning the concentration of degrading enzymes, or, if we rely on a PEN DNA toolbox implementation [25], through the use of the same enzyme for all species.

### 2.2. Simulation

We set n=2.4, $\alpha_{0,i}=0.1$, $\beta_{i}=1.0$, k=10.0. These parameters were selected in order to limit the range of the amplitudes of $m_{i,j}$ and $p_{i,j}$ while providing a broad oscillation area with respect to the other parameters. Furthermore, we set the overall activation rate of each oscillator close to each other. The activation rate of each oscillator is set as $\alpha_{2}=6.0$, $\alpha_{1}=\alpha_{2}-\gamma$, $\alpha_{3}=\alpha_{2}+\gamma$. The value of $\alpha_{2}$ was selected through a preliminary exploration of the behavior of the system, showing the emergence of complex structures (see Supplementary Figure S1). The initial values of $m_{i,j}$ and $p_{i,j}$ are randomized between 0 and 1.

### 2.3. Measure of Period and Synchronization

We used the autocorrelation function in order to calculate the overall period of the system. The normalized autocorrelation function with time lag kΔt is given by: [16]

(3) $$R_{m_{ij}m_{ij}}\left(k\right)=\frac{\frac{1}{N}\sum_{l=0}^{N-1}{\bar{m}}_{ij}\left(l\Delta t\right)\cdot {\bar{m}}_{ij}\left(\left(l+k\right)\Delta t\right)}{\frac{1}{N}\sum_{l=0}^{N-1}{\bar{m}}_{ij}\left(l\Delta t\right)\cdot {\bar{m}}_{ij}\left(l\Delta t\right)}$$

(4) $$\mathrm{with}\;{\bar{m}}_{ij}\left(l\Delta t\right)=m_{ij}\left(l\Delta t\right)-\frac{1}{N}\sum_{k=1}^{N}m_{ij}\left(k\Delta t\right)$$

where *N* is the number of samples. To measure a period of any of the oscillator, we look for the time lag that provides a strong autocorrelation (0.7 or above). Specifically, period $\tau_{i}={\tilde{k}}_{i}\Delta t$, where ${\tilde{k}}_{i}$ is the minimum $k_{i}$ which meets $R_{m_{ij}m_{ij}}\left(k_{i}\right)>R_{m_{ij}m_{ij}}\left(k_{i}-1\right)$, $R_{m_{ij}m_{ij}}\left(k_{i}\right)>R_{m_{ij}m_{ij}}\left(k_{i}+1\right)$ and $R_{m_{ij}m_{ij}}\left(k_{i}\right)>0.7$. In this paper, we consider the first element of any repressilator (j=1) to measure its period.

We then calculated the difference of periods Δp between oscillators to evaluate synchronization. Δp is defined as follows:

(5) $$\Delta p=\sum_{i>j}{\left(\tau_{i}-\tau_{j}\right)}^{2}$$

## 3. Result

**Figure 2:**
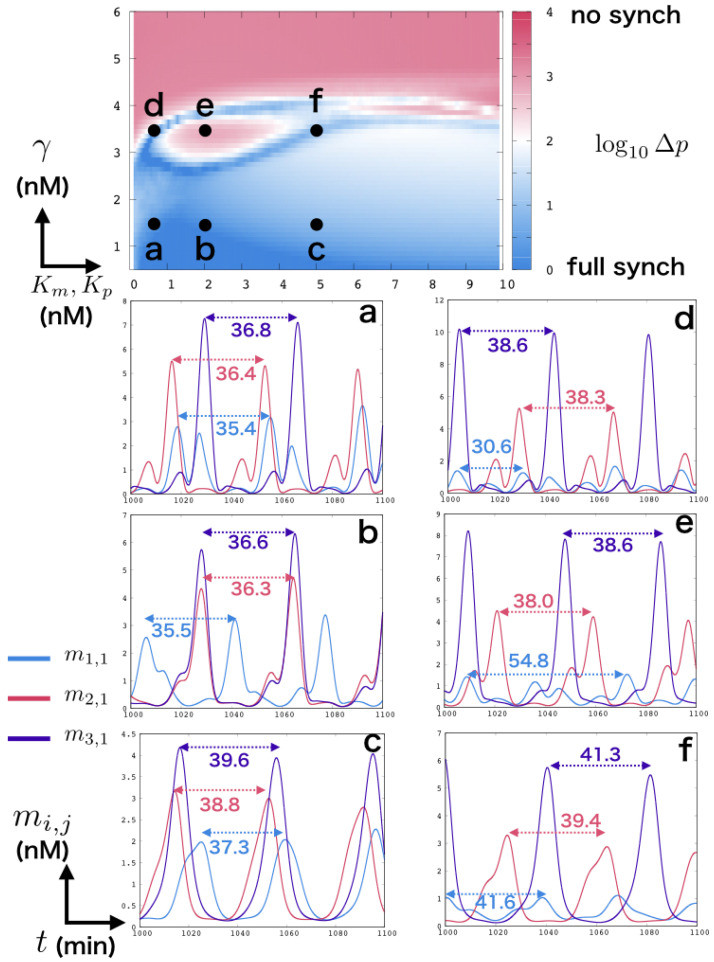
Top: Heatmap of synchronization in the system. The horizontal axis is $K_{m},K_{p}$ and the vertical axis is γ. The color is set by $\log_{10}\Delta p$. Bottom (**a**–**f**): time-series data for 6 points of interest in the parameter space. Light blue is $m_{1,1}$, red is $m_{2,1}$ and purple is $m_{3,1}$. Arrows indicate the respective period of the oscillators.

We measured the synchronization of the system over the parameter range of γ and $K_{m},K_{p}$. Figure 2, top, shows the results averaged over 100 simulations. For small values of γ, i.e., similar amplitude of oscillations, the repressilators are always at least partially synchronized. Indeed, regardless of coupling, the conditions of all three systems are near identical, leading to similar behaviors. As γ increases, the natural frequency of the three repressilators grow further apart, decreasing the overall synchronization of the system, until a sharp transition around γ=4.0 where the weakest repressilator cannot sustain oscillations on its own. At that point, the oscillator is instead driven by the rest of the system (Supplementary Figures S2–S6). As expected, for large values of $K_{m}$ and $K_{p}$, i.e., lower impact of competitive enzymatic saturation, the overall synchronization of the system tends to decrease. However, we can observe some unexpected behavior between 0.5 and 5 for $K_{m}$ and $K_{p}$, from 2.5 to 4 in γ (d, e, f in Figure 2), where an island of asynchronous behaviors is surrounded by strong coupled behaviors.

In order to further analyze the system, we rely on time-series data (Figure 2, bottom). By comparing (a) and (b) to (d) and (e), we can see that a small decrease in enzymatic coupling does not overall affect the period of the system. However, we can see that in the case of (e), $m_{1,1}$ seems to reach a different mode, with a period nearly doubled, which explains the sudden drop in overall synchronization of the system. This phenomenon can already be seen partially in (a) where $m_{1,1}$ has two distinct peaks, due to the individual influence of both of the other oscillators. As such, we expect the island to be created by the competition between the natural frequency of the weaker repressilator and the combined drive of the two other repressilators. When the impact of saturation is further decreased, in (c) and (f), interaction between oscillators are lighter, allowing the system to synchronize without affecting the shape of oscillations. Finally, for large values of γ, the periods and amplitudes of the oscillators are too different to allow for synchronization (Supplementary Figures S2–S6).

We can note that the shape of the oscillations is greatly affected by the enzymatic coupling. Even at low coupling levels (Figure 2c,f), the sharpness of peaks is decreased, and higher enzymatic coupling produces secondary peaks. Such results are similar to that of Fujii and Rondelez, who demonstrated in vitro the impact of enzymatic coupling on a system of two oscillators [14]. However, contrary to their results, we observe cases like (b) where two of the three oscillators are in phase. This phenomenon can be explained by the fact that the oscillator with the highest production rate (repressilator 3 in the case of (b)) saturates the degradation enzyme, thus buffering the other two oscillators and allowing them to spike. Due to the lack of direct coupling, however, there is no constraint on which species of the respective oscillators will get in phase. In the case of Figure 2b, $m_{1,3}$, $m_{2,1}$, and $m_{3,1}$ are in phase.

Finally, we performed a spectral analysis of the system (see Supplementary Materials) which highlighted the natural frequency of each oscillators (dependent on their activator concentration) and the additional frequency due to the impact of enzymes.

## 4. Conclusions

In this paper, we showed that indirect interaction through competition for enzymes between independent molecular oscillator could lead to their synchronization, as long as their impact on enzymes are similar. An interesting phenomenon arises when the activation mechanism of a weaker oscillator is prevented by the saturation of enzymes by the stronger ones, leading to a sharp transition to an almost doubled period. Those results can be contrasted with directly coupled repressilators, in which the parameter plane of production versus coupling shows a transition between synchronization and chaotic behaviors [7].

Molecular implementation of the system can be done directly with the PEN DNA toolbox framework [5,6], as it allows to produce a wide range of reaction networks. In particular, an equivalent of the Repressilator has been implemented by Padirac [25]. As the PEN DNA toolbox offers flexibility in the design of molecular species, that design can be extended to produce three independent identical networks, as used in the present article. The current exploration can also be directly applied to other types of molecular oscillators, such as the molecular predator-prey [14], the Oligator [5], or even combinations of different types of oscillators as long as they share the same enzymes. In all cases, while we do expect a major reality gap between the model and experimental results, that gap can be bridged by sampling thousands of candidate parameters in vitro through a specialized microfluidic platform [26].

Finally, indirect coupling in molecular robotics, is usually seen as having a negative impact on the system as those are more non-linear and thus harder to control. However, our results show that, in this case, it provides a complex behavioral space allowing us to sharply transition between different coupling modes through small variations in enzymatic saturation (Figure 2d–f). In particular, enzymatic activity can be directly modified by changing the temperature of the environment [17]. By setting the system near the transition area shown in our results, we may force a system in and out of synchronization over temporal or spatial patterns.

Systems made of a single molecular oscillator have been used to perform computation [17], control the swarming of molecular robots [27], and produce reaction–diffusion patterns [28]. As those systems all rely on enzymatic activity, we expect that increasing the number of oscillators will provide a much richer repertoire of behaviors. As such, potential applications range from developing controllers for molecular robots, where each oscillator affects a different type of robot, to designing active materials, locking in areas where multiple oscillators peak at the same time. 

## Data Availability

Code is available at https://github.com/shihoinagaki/repressilator_cross_saturation.

## Supplementary Materials

The following are available online at https://www.mdpi.com/article/10.3390/mi13020245/s1, Figure S1: coarse-grained exploration of $\alpha_{2}$, Figures S2–S6: autocorellation function and power spectrum for parameters of interest.
