# Supplementary material for: Controlling the Synchronization of Molecular Oscillators through Indirect Coupling
Source: Micromachines (Basel). 2022 Feb 1;13(2):245. doi: 10.3390/mi13020245 (PMC8877793; doi:10.3390/mi13020245)
Supplement: Supplementary file 1 [file micromachines-13-00245-s001.zip › micromachines-1531749-supplementary.pdf]

Supplementary materials: Controlling The Synchronization of Molecular Oscillators Through Indirect Coupling

Shiho Inagaki and Nathanael Aubert-Kato

January 26, 2022

1 Coarse-grained exploration of α_2

As a preliminary analysis of the 3 repressilators system, we first evaluated a coarse-grained mapping for $\alpha_2 = 5.0$, $\alpha_2 = 6.0$, and $\alpha_2 = 7.0$ (Supplementary Figure 1). When $\alpha_2 = 5.0$, there are only two regions, corresponding to synchronization and no synchronization, respectively. The transition occurs around $\alpha_2/2$, regardless of enzymatic saturation. For both $\alpha_2 = 6.0$ and $\alpha_2 = 7.0$, more complex structures emerged near the transition area. However, for $\alpha_2 = 7.0$, the island looked smaller than that of $\alpha_2 = 6.0$. If α_2 is large, the system should be easier to synchronize even if γ is large, which might explain that difference. For those reason, we eventually decided to pick $\alpha_2 = 6.0$ for the full exploration.

2 Power spectrum calculation

We calculated power spectrum of the repressilator systems to confirm the period of the different oscillators. First, discrete Fourier-transformation is performed on $\bar{m}_{ij}$, which is equal to m_{ij} minus its time average.

$$M(\mu) = \sum_{k=0}^{N-1} m_{ij} \exp^{-\mathrm{i}\frac{2\pi\mu k}{N}}$$

The power spectrum is then calculated as

$$PS(\mu) = Re(M(\mu))^2 + Im(M(\mu))^2$$

It is then normalized as PSD so that the sum is equal to 1.

$$PSD(\mu) = \frac{PS(\mu)}{\sum_{\mu=1}^{N} PS(\mu)}$$

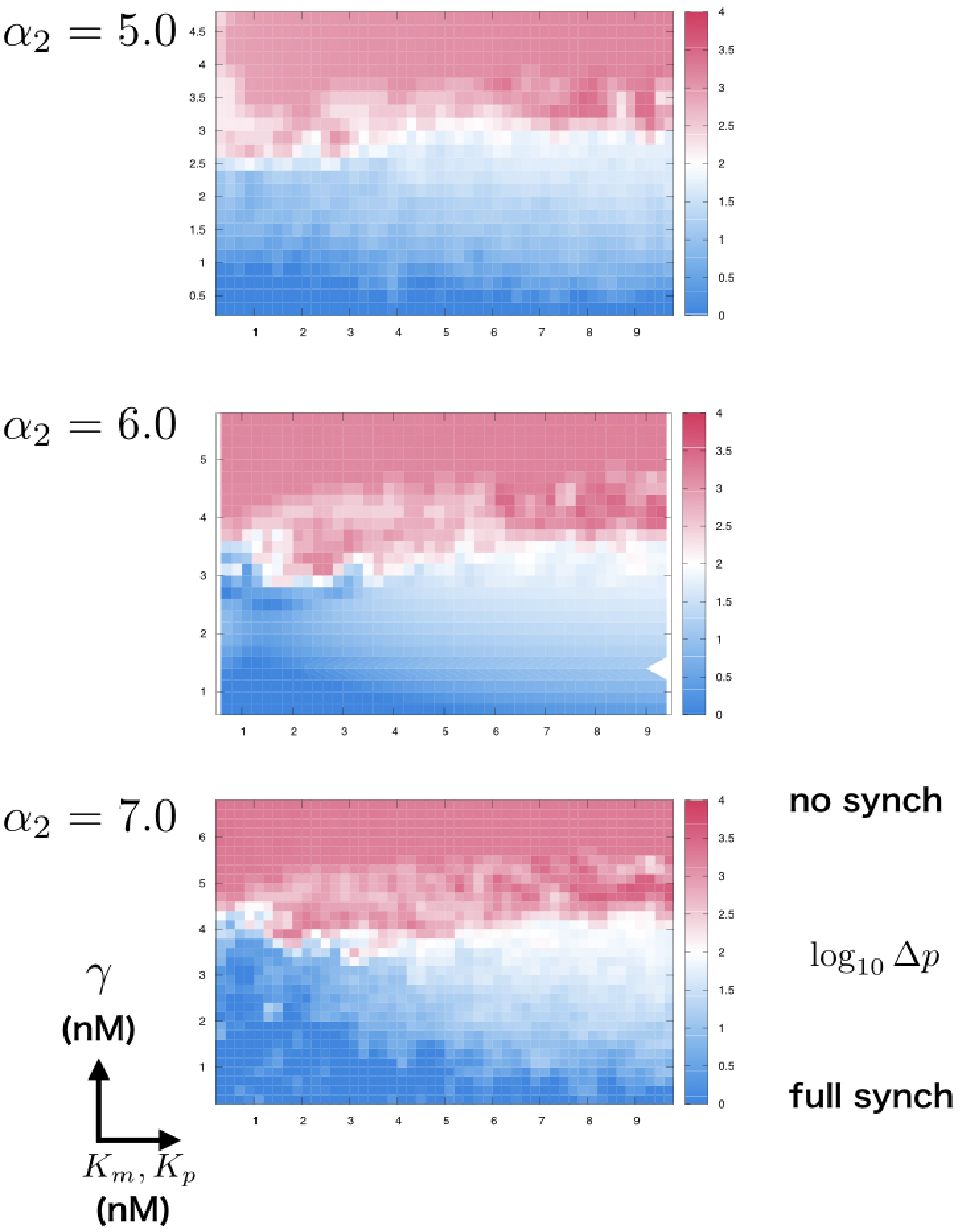

Figure S1: Phase map of top: $\alpha_2 = 5.0$, bottom: $\alpha_2 = 7.0$.

3 Analysis of points of interest

The following figure plots the time series data, autocorrelation function, and power spectrum of the repressilators for relevant combinations of parameters. The arrow indicates the average period over 100 evaluation, but plots are given for a single simulation. For power spectrum figures, the horizontal axis represents the period, which is the reciprocal to the frequency. The autocorrelation function of $m_{1,1}$ for (d) and (e) shows that there are two nearly identical peaks around 30 and 55. The period of the repressilator changes abruptly once the latter peak overcomes the former.

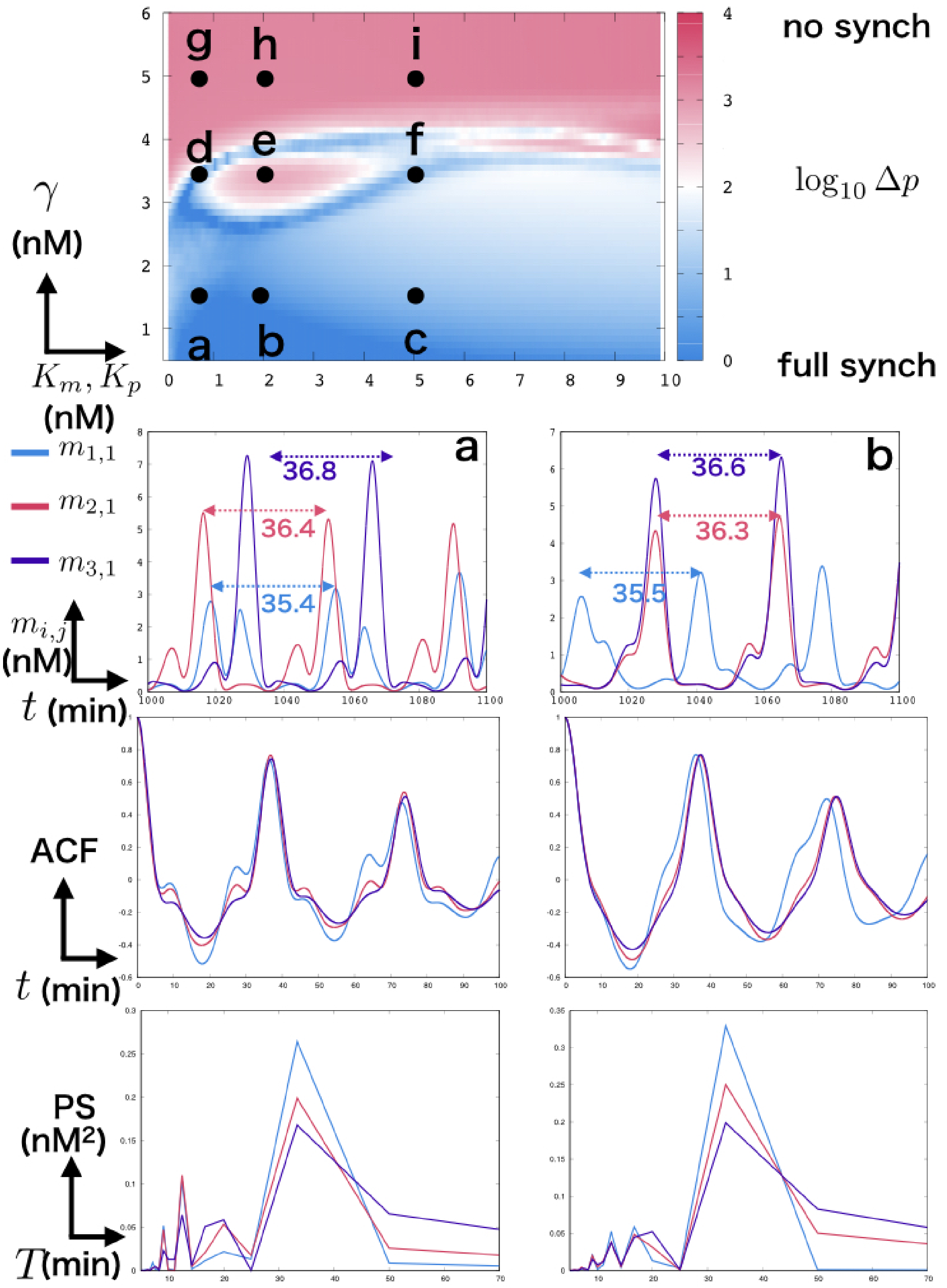

Figure S2: Top: time-series, middle: autocorrelation function, bottom: power spectrum. The left:$K_m, K_p = 0.5$, $\gamma = 1.5$. The right:$K_m, K_p = 2.0$, $\gamma = 1.5$

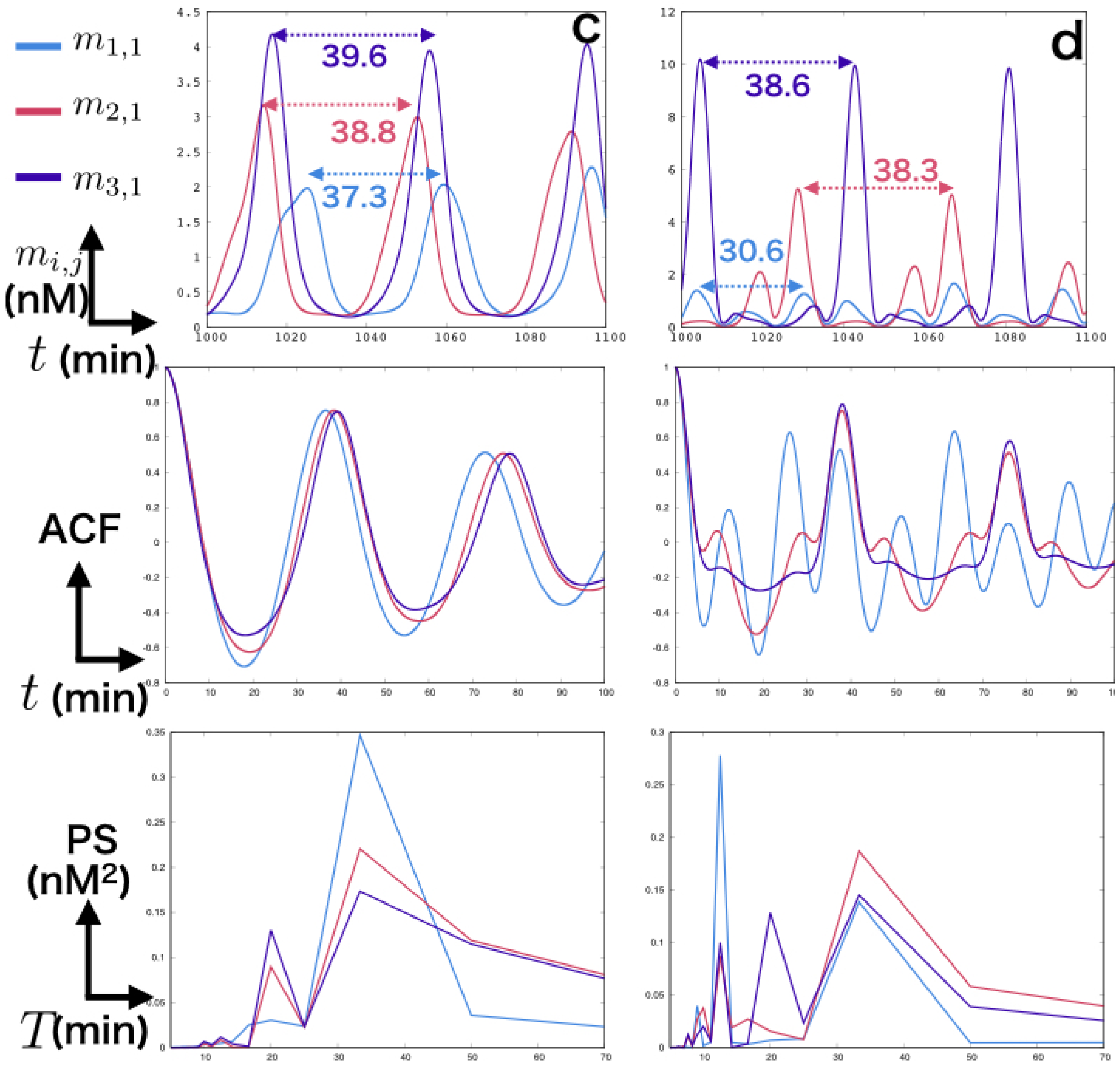

Figure S3: Top: time-series, middle: autocorrelation function, bottom: power spectrum. The left:$K_m, K_p = 5.0$, $\gamma = 1.5$. The right:$K_m, K_p = 0.5$, $\gamma = 3.5$

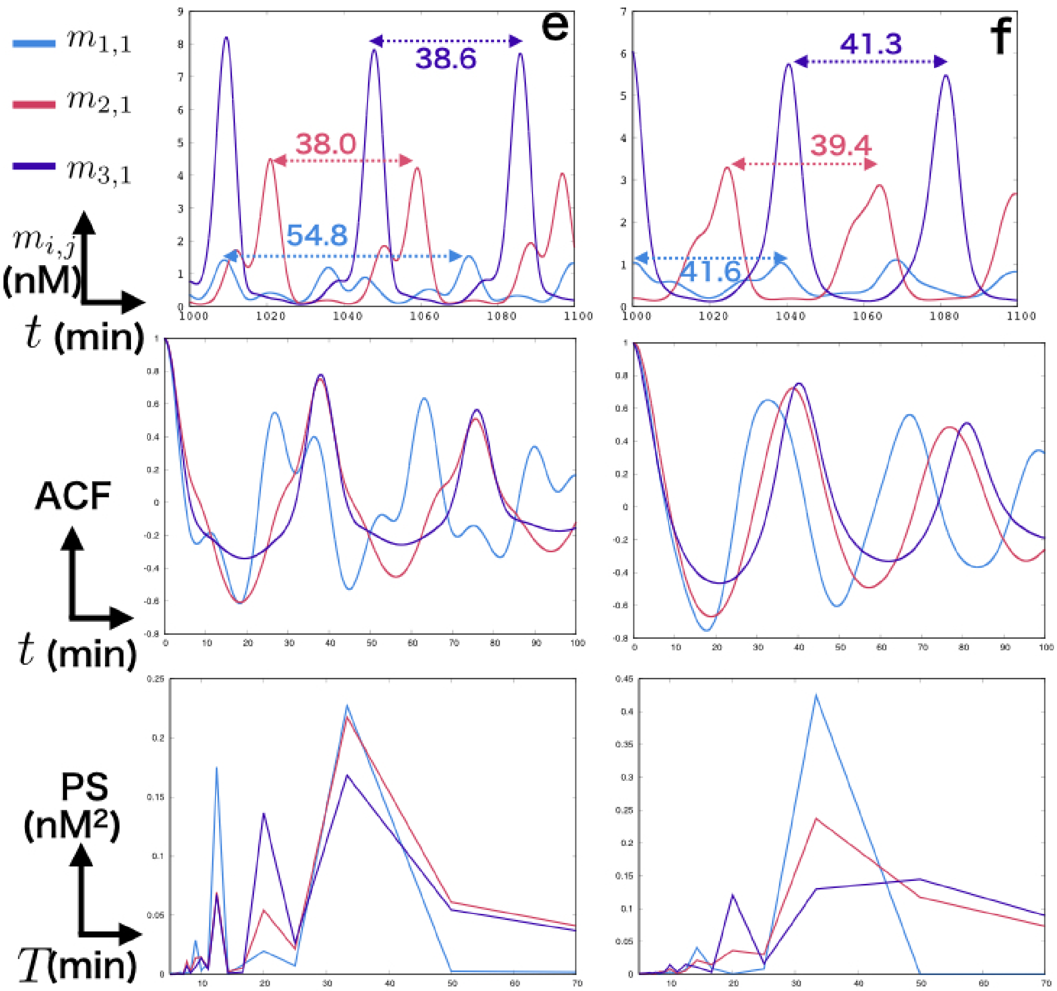

Figure S4: Top: time-series, middle: autocorrelation function, bottom: power spectrum. The left:$K_m, K_p = 2.0$, $\gamma = 3.5$. The right:$K_m, K_p = 5.0$, $\gamma = 3.5$

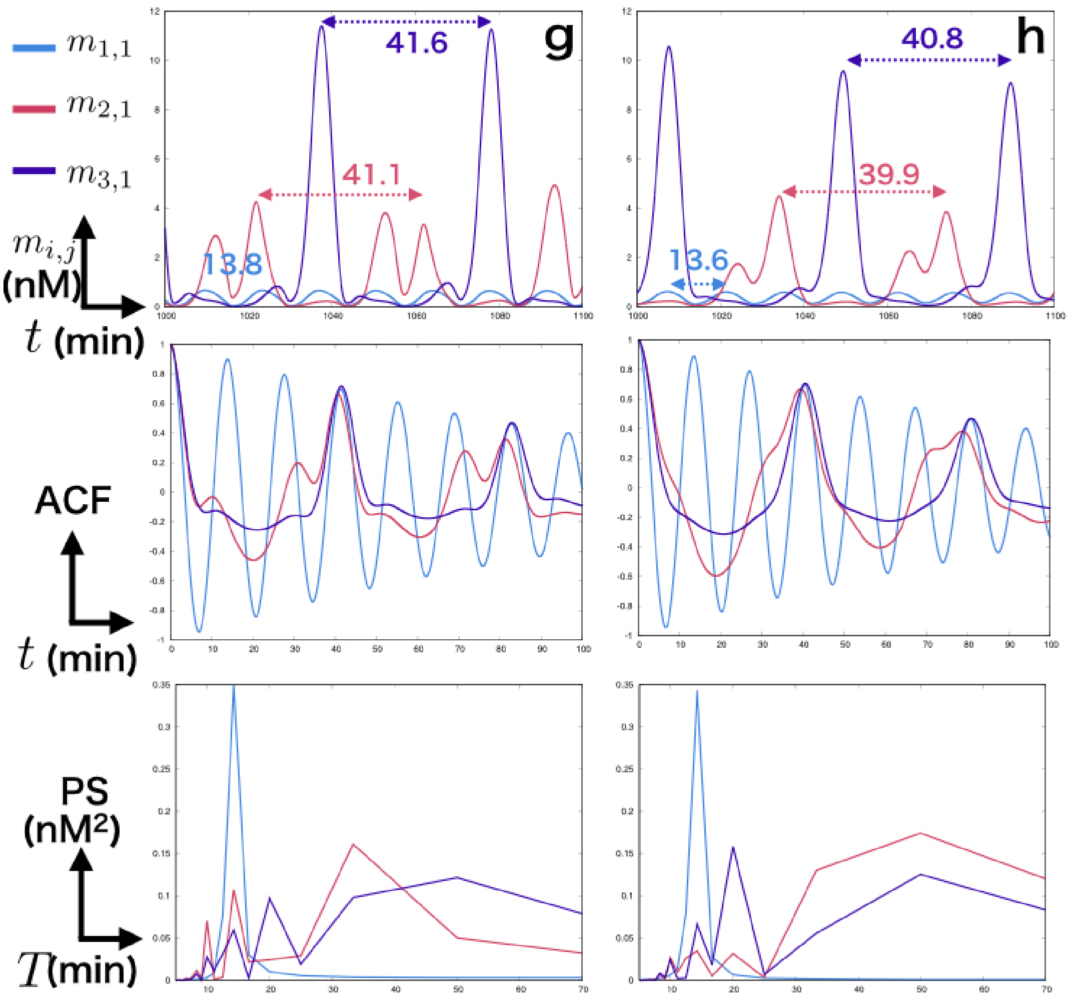

Figure S5: Top: time-series, middle: autocorrelation function, bottom: power spectrum. The left:$K_m, K_p = 0.5$, $\gamma = 5.0$. The right:$K_m, K_p = 2.0$, $\gamma = 5.0$

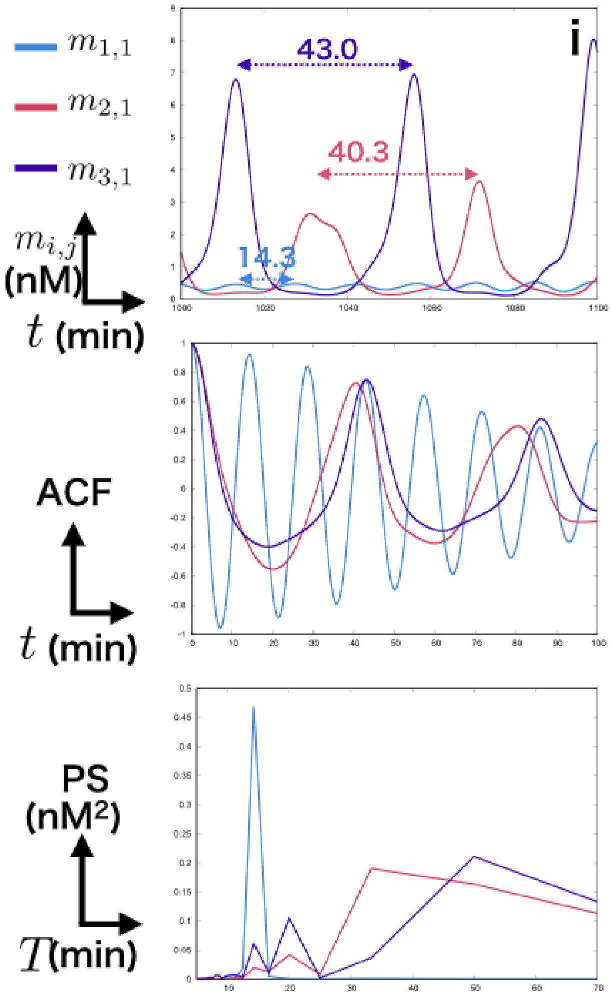

Figure S6: Top: time-series, middle: autocorrelation function, bottom: power spectrum. K_m, $K_p = 2.0$, $\gamma = 5.0$.
